# Subtrochanteric fracture after femoral neck system of femoral neck fractures: a report of four cases

**DOI:** 10.1186/s12891-023-06872-2

**Published:** 2023-09-22

**Authors:** John C. Fisher, Christopher Gerzina, Kaitlin Rush, Cyrus Caroom

**Affiliations:** Texas Tech University Health Sciences Center (TTUHSC), 3601 4th St., Lubbock, Tx 79430 USA

**Keywords:** Femoral neck fracture, Femoral neck system, Peri-implant fracture, Complication of hip fracture treatment, Trauma, Femoral neck fixation, Open reduction internal fixation femoral neck, Proximal femur fracture, Subtrochanteric femur fracture

## Abstract

**Background:**

The femoral neck system (FNS) is commonly used for internal fixation of femoral neck fractures and has shown promising results. However, we have observed cases of peri-implant subtrochanteric femur fractures associated with the use of FNS at our institution. This case series aims to investigate four cases of peri-implant subtrochanteric fractures in patients treated with the FNS implant for femoral neck fractures.

**Case presentation:**

We reviewed 35 patients who underwent treatment with FNS for femoral neck fractures between January 2017 and December 2021 at our level 1 trauma institution. Among these patients, four cases of peri-implant subtrochanteric femur fractures were identified. In contrast, no such fractures occurred in patients treated with cannulated screws or dynamic hip screws (DHS). Interestingly, all four cases of peri-implant fractures were seen in patients with incomplete nondisplaced femoral neck fractures. Only one case involved an identifiable technical error.

**Conclusions:**

This case series sheds light on peri-implant subtrochanteric femur fractures as a previously unreported complication associated with the use of FNS for femoral neck fractures. These fractures were observed exclusively in patients with incomplete nondisplaced fractures who received FNS fixation. No similar complications were observed in patients treated with other types of fixation. This finding suggests the need for caution and further investigation when considering FNS as a treatment option for this specific fracture pattern.

The identification of peri-implant subtrochanteric femur fractures as a potential complication of FNS usage in incomplete nondisplaced femoral neck fractures raises important considerations for clinical decision-making and patient management in orthopedic trauma.

## Background

Femoral neck fractures present a challenge for the treating orthopedic surgeon, and various fixation methods are used. The femoral neck system (FNS) is a newer implant that has shown promising results in early biomechanical studies [1–7]. However, there have been reports of subtrochanteric femur fractures associated with the FNS. In this series, we discuss four cases of peri-implant subtrochanteric fractures after implantation of the FNS.

Peri-implant sub trochanteric fractures have been observed in cannulated screw fixation of femoral neck fractures and are believed to be due to a stress riser caused by placing screws distal to the lesser trochanter [8–10]. We suspect the FNS has an increased risk of similar fractures, but the reason is unclear. This series discusses four cases of peri-implant subtrochanteric fractures after implantation of FNS observed over four years of use.

## Case presentation

### Case 1

**Fig. 1:**
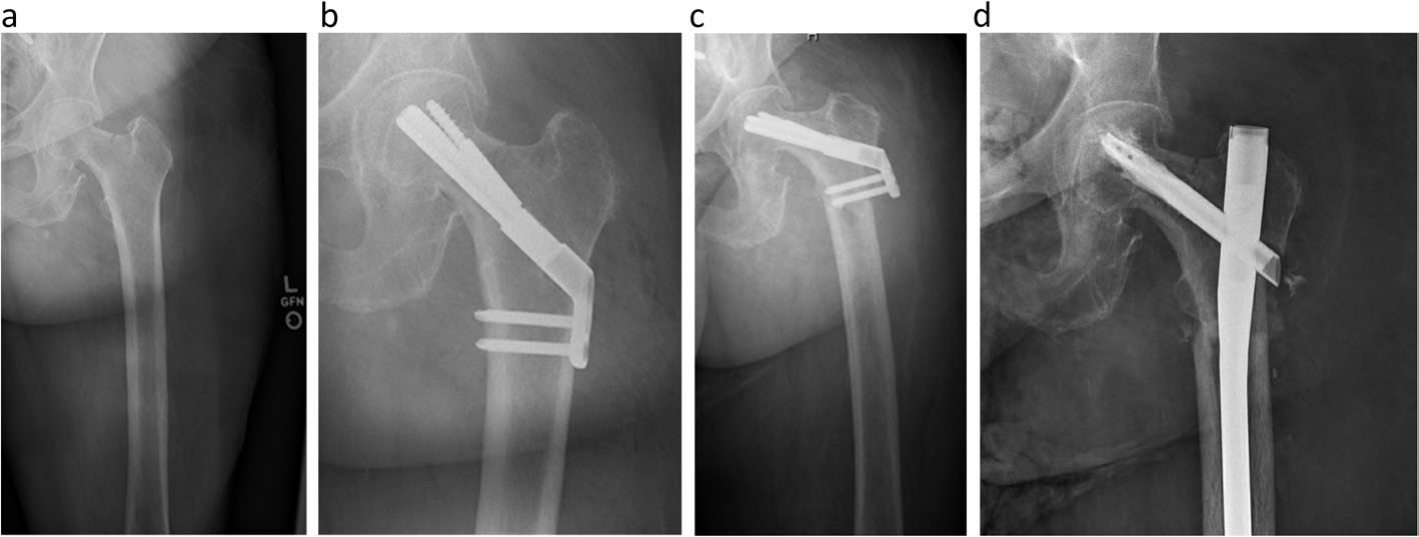
68-year-old female with 1 week of progressive hip pain. Panel **a** is the injury AP radiograph of the left hip showing an incomplete non-displaced fracture. Panel **b** are postoperative AP radiographs of the left hip showing the implanted femoral neck system. Panel **c** is an AP radiograph of the left hip taken 41 days postoperatively revealing a peri-implant subtrochanteric fracture. Panel **d** is an AP radiograph of the left hip taken at 6-week postoperative visit showing successful treatment with a cephalomedullary nail

A 68-year-old woman with a medical history of hypertension, coronary artery disease, hypothyroidism, and alkaptonuria was diagnosed with a Garden I fracture after a ground-level fall three weeks before presentation. She reported mild pain in the hip, described as acute on chronic pain in the extremity. The patient underwent internal fixation using a FNS and was allowed to immediately bear weight. She was discharged on postoperative day three to a skilled nursing facility. However, six weeks postoperatively she fell again and was unable to bear weight. A peri-implant subtrochanteric femur fracture at the level of the distal screw was discovered on radiographs, and she was successfully treated with placement of a reamed cephalomedullary nail with cement augmentation. (Fig. 1).

### Case 2

**Fig. 2:**
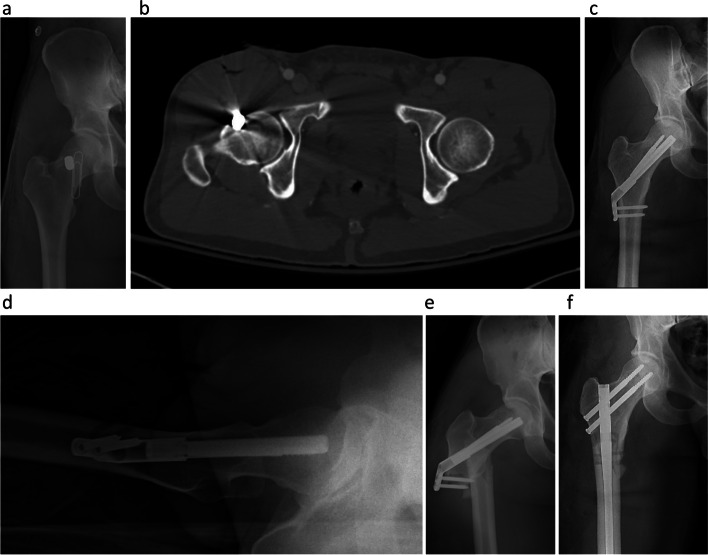
**a** 41-year-old male presented to our institution after a gunshot wound to his right groin. Panel **a** is an AP radiograph of the right hip showing a foreign body overlying the femoral neck. Paper clip denotes the entry wound of the missile. Panel **b** is an axial cut from computed tomography of the right hip showing an intracapsular missile with disruption of the anterior cortex of the femoral neck. Panel **c** is a postoperative anteroposterior and **d** lateral radiograph of the right hip after implantation of a femoral neck system showing unfilled screw holes in the lateral cortex. Panel **e** is an AP radiograph of the right hip 17 days post-operatively revealing a peri-implant subtrochanteric fracture. He was treated successfully with a piriformis entry recon nail shown in panel **f**

A 41-year-old man with no medical history presented after a gunshot wound to the right proximal femur. Radiographs showed a retained missile, and computed tomography revealed an incomplete, nondisplaced femoral neck fracture involving the anterior cortex of the femur with a retained intracapsular bullet. The patient underwent fixation with an FNS system and was discharged on postoperative day two after an uneventful postoperative course. However, he sustained a peri-implant subtrochanteric femur fracture after a ground-level fall 17 days postoperatively. The patient was treated with a reamed piriformis entry recon nail. Upon critical review of the case, it was discovered that the lateral cortex screws had been replaced to reposition the plate, leaving two unfilled drill holes seen on immediate postoperative radiographs. (Fig. 2).

### Case 3

**Fig. 3:**
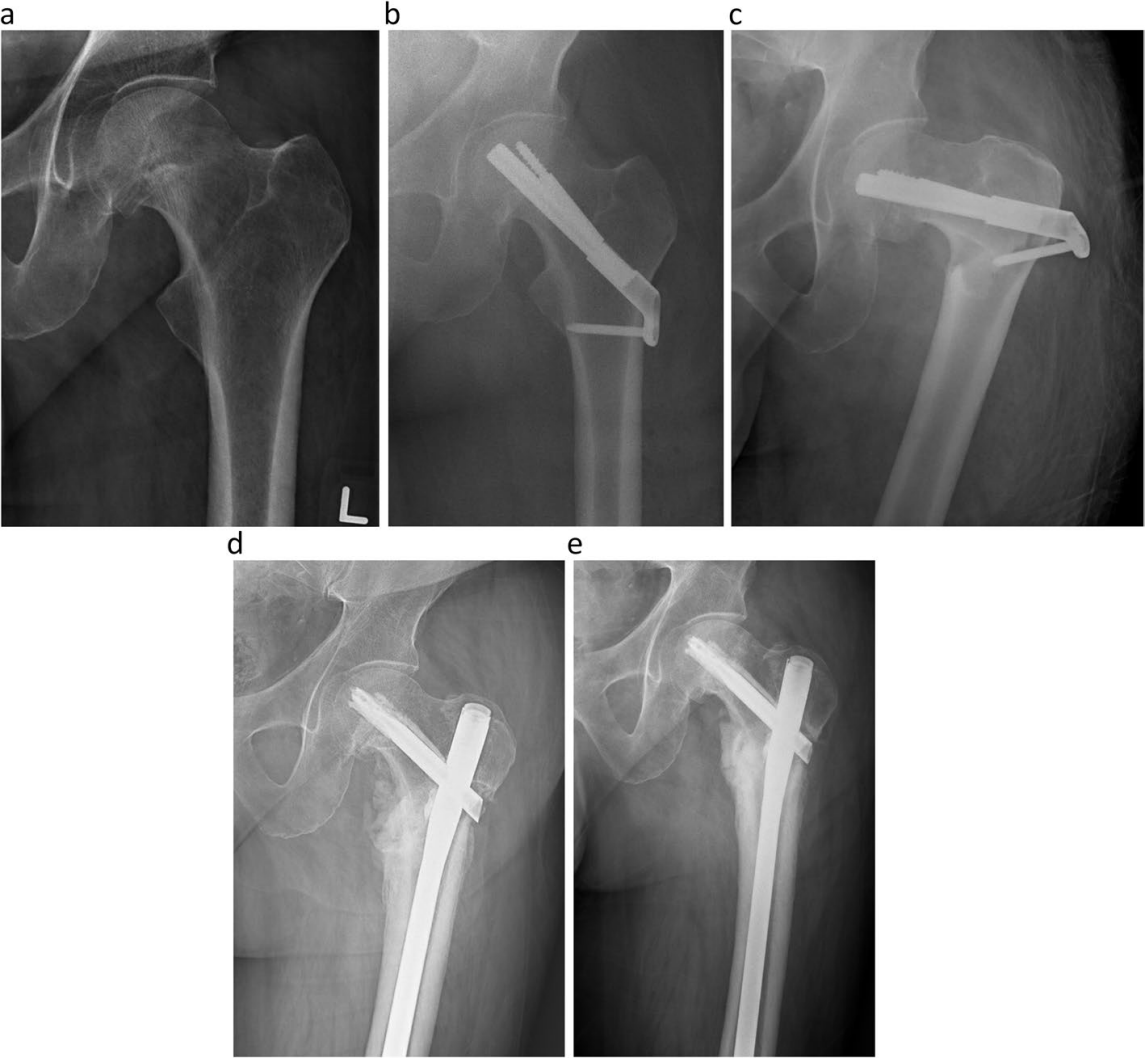
Panel **a** is an anteroposterior radiograph of the left hip showing an incomplete nondisplaced fracture of the femoral neck on the tension side of the femoral neck. Panel **b** is an anteroposterior radiograph of the hip after successful implantation of FNS. Panel **c** is an anteroposterior radiograph of the left hip showing a peri-implant subtrochanteric fracture at the level of the distal screw. Panel **d** is an anteroposterior radiograph of the left hip taken 9 months after implantation of cephalomedullary nail with painful nonunion. Panel **e** is a radiograph taken 1 year postoperatively after removal of distal interlocking screws showing callus formation

A 65-year-old man with a past medical history of hypertension and cardiac arrhythmia with a pacemaker presented with insidious onset hip pain that had worsened over the course of a day, necessitating the use of a walker for ambulation. Radiographs revealed a tension-side, incomplete, nondisplaced femoral neck fracture. The patient underwent fixation with an FNS. Six weeks later, he sustained a ground-level fall and was found to have a peri-implant subtrochanteric fracture at the level of the distal screw. The patient was treated with a reamed cephalomedullary nail, which subsequently went on to nonunion. The patient successfully underwent revision one year later with removal of the distal interlocking screws. (Fig. 3).

### Case 4

**Fig. 4:**
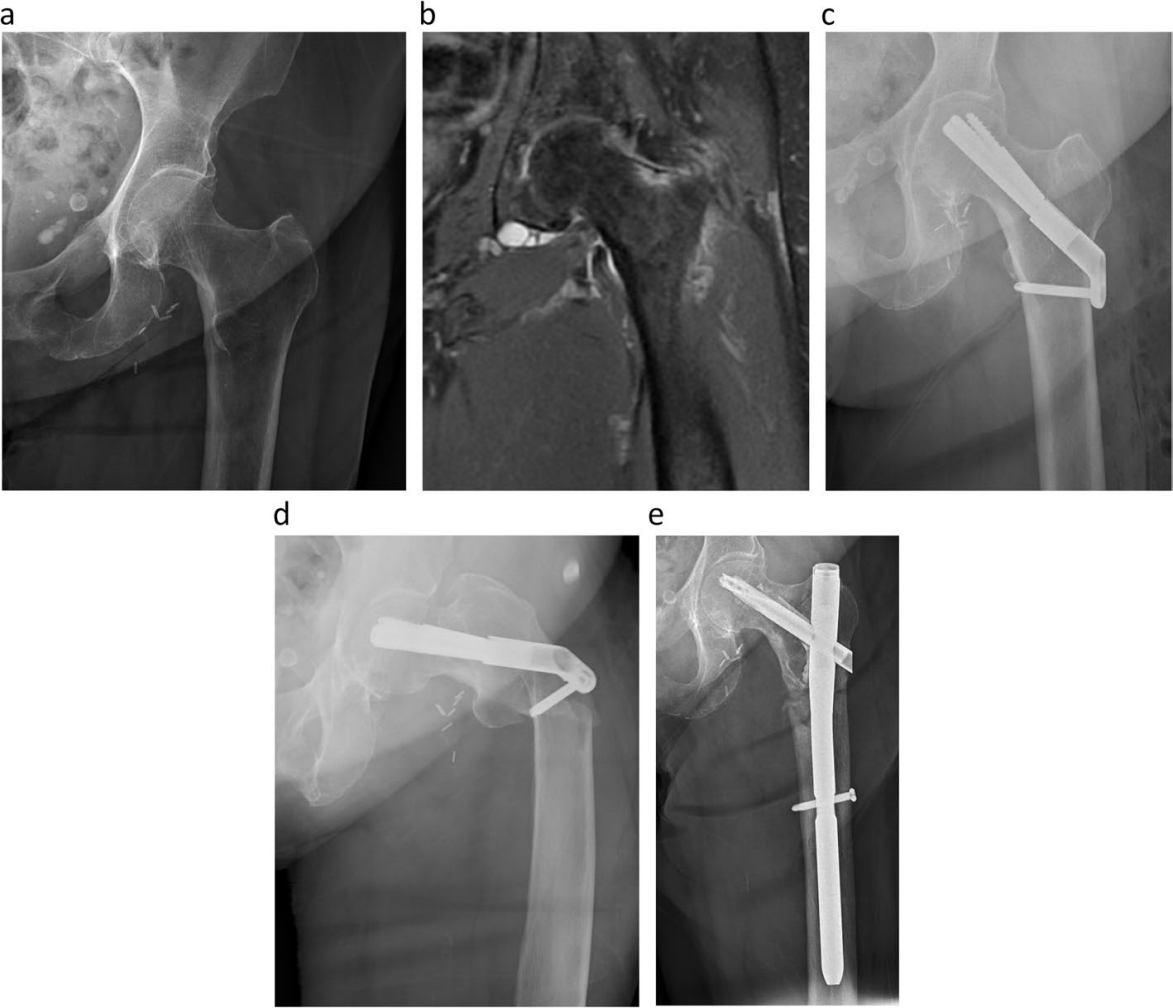
Panel **a** is an anteroposterior radiograph of the hip showing an incomplete nondisplaced fracture on the tension side of the femoral neck. Panel **b** is a coronal T2 fat suppressed MRI showing hyperintense signal on the tension side of the femoral neck. Panel **c** is an anteroposterior hip radiograph showing successful implantation of an FNS. Panel **d** is an anteroposterior hip radiograph showing peri-implant subtrochanteric femur fracture at the distal screw hole of the FNS. Panel **e** is an anteroposterior radiograph of the hip showing successful treatment with an intermediate cephalomedullary nail taken three months postoperatively

A 74-year-old female with a past medical history of osteoporosis, end-stage renal disease, deep venous thrombosis with pulmonary embolism, cerebral vascular accident, and peptic ulcers presented with a femoral neck stress fracture. Her contralateral hip was successfully treated for a stress fracture three years ago with three cannulated screws. She had months of antecedent pain in her left hip and was treated with an FNS. At her request, she was discharged home with home health on postoperative day one. Three weeks later, she returned with an insidious onset of left hip pain and an inability to bear weight. Radiographs showed a peri-implant subtrochanteric femur fracture at the level of the distal screw. The time to failure was 21 days. The patient was successfully treated with a cephalomedullary nail. (Fig. 4).

## Discussion and conclusions

Peri-implant subtrochanteric fractures are a well-known complication of cannulated screw fixation, which tends to occur when screws are placed distal to the lesser trochanter. Studies have shown that screws placed in this region are associated with an increased risk of peri-implant subtrochanteric fracture [8–10]. Our institution started using one-hole lateral plates to place the distal screw proximal to the lesser trochanter whenever possible. As shown in Figs. 3 and 4, patients with one-hole plates were not spared of this complication.

The FNS design is characterized by locking screws in the plate, which increases construct stiffness and is hypothesized to contribute to peri-implant subtrochanteric fracture. In contrast, the SHS features cortical screws that are not locked into a lateral cortex plate. Over the period of observation, no peri-implant fractures occurred in patients who received SHS. Further study is required to explore this aspect in more detail.

Notably, all our cases involved patients with incomplete nondisplaced femoral neck fractures on the tension side of the bone who were treated with the FNS. We made a distinction in the Garden I femoral neck fractures between valgus impacted patterns and incomplete nondisplaced femoral neck fractures when reviewing our cases. We identified eight patients with incomplete nondisplaced femoral neck fractures on the tension side of the neck during our use of the FNS from 2017 to 2021.

Among these eight patients, seven had histories consistent with stress fractures, while one patient sustained a gunshot injury to the femoral neck (Fig. 2). Treatment varied for these patients. Six received an FNS, of which four went on to peri-implant subtrochanteric femur fracture. One received SHS, and one received 3CS, both of which healed without event.

The suggested association between the incomplete non-displaced fracture pattern and peri-implant subtrochanteric fractures has an unknown cause. The authors believe that the stress-fracture pattern implicates poor bone quality throughout the femur and magnifies the stress riser effect on the lateral cortex of the femur, but further biomechanical investigation is needed.

One of the treated patients had multiple holes drilled in the proximal femoral cortex for better placement of the cortical plate (Fig. 2). The subsequent fracture through the unfilled screw hole supports the theory that stress risers created by multiple holes may contribute to the development of peri-implant subtrochanteric fractures. It is important for surgeons to use proper techniques to avoid unnecessary violation of the femoral cortex.

Several studies have demonstrated the favorable biomechanical properties of the FNS. In one such study, Stoffel et al. compared the biomechanical performance of the FNS with 3CS and DHS in 20 cadaveric models. While no difference was found in mean axial stiffness between the groups, 3CS showed significantly fewer cycles until 15 mm of shortening of the femoral neck and the leg compared to both FNS and DHS groups. Overall, this study suggests that the FNS exhibits favorable biomechanics compared to 3CS in the treatment of femoral neck fractures [1].

Studies reporting clinical outcomes of FNS continue to affirm the safety and efficacy of the FNS. Davidson et al. conducted a retrospective study of 102 patients and reviewed the literature, including 278 patients, reporting a 9.2% revision rate, and concluding that FNS is a safe treatment option for femoral neck fractures [11]. Jiang et al. performed a systematic review and meta-analysis that encompassed eight studies with 448 patients. The analysis revealed that FNS was associated with lower radiation exposure, decreased fracture healing time, reduced femoral head necrosis, fewer implant failures/cutouts, lower visual analog scale scores, and higher Harris hip scores. The authors conclude that FNS is an effective choice for treating femoral neck fractures [12].

Rajnish et al. conducted a systematic review and meta-analysis of six retrospective studies involving 371 young adults aged 18–65 years who were treated with FNS for femoral neck fractures. The analysis revealed that FNS resulted in significantly lower fluoroscopy time and higher blood loss than the 3CS group. However, no significant differences were found in surgery time, incision length, length of hospital stay, complications, pain relief, or functional outcomes. As a result, the study concluded that there were similar outcomes between the FNS and 3CS groups in the young adult population [13].

Vazquez et al. conducted a retrospective review to assess short-term outcomes in Garden I and II femoral neck fractures in patients aged 75 years or older who were treated with FNS, DHS, and 3CS. The study found a significantly lower duration of surgery in the FNS group, with similar impaction and shortening observed between the groups. This evidence supports the use of FNS for treating femoral neck fractures in elderly patients due to the reduced surgical duration [2].

Additionally, Nibe et al. retrospectively examined clinical outcomes in 52 patients aged 65 years and older with femoral neck fractures, of which 25 were treated with FNS and 27 with other implants. The FNS group showed shorter operative times, lower reoperation rates, and a 100% union rate. No significant difference in average blood loss was observed. Based on these findings, the study concludes that FNS represents a viable alternative for treating femoral neck fractures in an elderly population due to its shorter surgical times and lower reoperation rates [5].

Femoral neck fractures are commonly managed with internal fixation methods, and the FNS is a newer option. However, our cases demonstrate that peri-implant subtrochanteric fractures can occur in patients with incomplete nondisplaced femoral neck fractures treated with the FNS. Proper surgical techniques and minimizing violations of the femoral cortex are crucial in preventing these fractures. Further studies are needed to explore the association between the FNS design and the risk of subtrochanteric fractures.

## Data Availability

The datasets used and/or analyzed during the current study are available from the corresponding author on reasonable request.

## Abbreviations

FNS: Femoral neck system
CMN: Cephalomedullary nail
3CS: Cannulated screw fixation
SHS: Sliding hip screw
